# Decision Forest Analysis of 61 Single Nucleotide Polymorphisms in a Case-Control Study of Esophageal Cancer; a novel method

**DOI:** 10.1186/1471-2105-6-S2-S4

**Published:** 2005-07-15

**Authors:** Qian Xie, Luke D Ratnasinghe, Huixiao Hong, Roger Perkins, Ze-Zhong Tang, Nan Hu, Philip R Taylor, Weida Tong

**Affiliations:** 1 Division of Bioinformatics, Z-tech at FDA's National Center for Toxicological Research, Jefferson, AR 72079; 2 Center for Structural Genomics, DPME, NCTR, Food and Drug Administration, Jefferson, AR 72079; 3 Shanxi Cancer Hospital, Taiyuan, Shanxi 030013, the Peoples Republic of China; 4 Genetic EpidemiologyBranch, DCEG, National Cancer Institute, Rockville, MD 20852; 5 Center for Toxicoinformatics, Division of Systems Toxicology, FDA's National Center for Toxicological Research, Jefferson, AR 72079

## Abstract

**Background:**

Systematic evaluation and study of single nucleotide polymorphisms (SNPs) made possible by high throughput genotyping technologies and bioinformatics promises to provide breakthroughs in the understanding of complex diseases. Understanding how the millions of SNPs in the human genome are involved in conferring susceptibility or resistance to disease, or in rendering a drug efficacious or toxic in the individual is a major goal of the relatively new fields of pharmacogenomics. Esophageal squamous cell carcinoma is a high-mortality cancer with complex etiology and progression involving both genetic and environmental factors. We examined the association between esophageal cancer risk and patterns of 61 SNPs in a case-control study for a population from Shanxi Province in North Central China that has among the highest rates of esophageal squamous cell carcinoma in the world.

**Methods:**

High-throughput Masscode mass spectrometry genotyping was done on genomic DNA from 574 individuals (394 cases and 180 age-frequency matched controls). SNPs were chosen from among genes involving DNA repair enzymes, and Phase I and Phase II enzymes.

We developed a novel adaptation of the Decision Forest pattern recognition method named Decision Forest for SNPs (DF-SNPs). The method was designated to analyze the SNP data.

**Results:**

The classifier in separating the cases from the controls developed with DF-SNPs gave concordance, sensitivity and specificity, of 94.7%, 99.0% and 85.1%, respectively; suggesting its usefulness for hypothesizing what SNPs or combinations of SNPs could be involved in susceptibility to esophageal cancer. Importantly, the DF-SNPs algorithm incorporated a randomization test for assessing the relevance (or importance) of individual SNPs, SNP types (Homozygous common, heterozygous and homozygous variant) and patterns of SNP types (SNP patterns) that differentiate cases from controls. For example, we found that the different genotypes of SNP GADD45B E1122 are all associated with cancer risk.

**Conclusion:**

The DF-SNPs method can be used to differentiate esophageal squamous cell carcinoma cases from controls based on individual SNPs, SNP types and SNP patterns. The method could be useful to identify potential biomarkers from the SNP data and complement existing methods for genotype analyses.

## Background

The cause and progression of human diseases such as Alzheimer's disease, cancer and diabetes are likely influenced by complex interactions of multiple genes as well as environmental and lifestyle factors that are sensitive to genome variability. Single nucleotide polymorphisms (SNPs) are the most prevalent form of DNA variation in the human genome occurring about once per 100 to 300 bases [1]. The high frequency of relatively stable SNPs makes them excellent biomarkers for some disease phenotypes. While some serious diseases such as cystic fibrosis and sickle cell anemia are of predominately genetic etiology in homozygote individuals, other serious diseases are far more complex. In more complex diseases, a combination of multiple SNPs (i.e., SNP patterns), plus environmental factors, may combine to determine disease susceptibility and prognosis. The terminology of "complex disease" is used because of the potential enormity of interacting genes that could be in the hundreds. The genetic factors that determine disease phenotypes may be encoded in the pattern of genomic variation that is primarily SNPs. Cancer, mental illness, some autoimmune disorders and diabetes are among the common serious diseases thought to be significantly influenced by the spectrum of SNPs an individual has in certain susceptibility genes. Population variability in drug response is thought to be analogously dependent on an individual's SNP profile.

Therefore, the identification of particular SNP patterns that are associated with susceptibility to disease or adverse drug reactions is a paramount goal of pharmacogenomics research. Given recent development of high-throughput technologies for rapid genotyping, assessment of SNP patterns holds much promise for use in diagnosis, prognosis and selection of treatment intervention. An essential bioinformatics challenge in pharmacogenomics is the discovery of SNP patterns that differentiate diseased and healthy populations.

Determination of the SNP patterns associated with disease susceptibility or adverse drug reaction from among potentially millions of SNPs is a challenge for pharmacogenomics. Many methods have been described in the literature to assess the association between SNPs and disease [2-5]. Most of them are focused on identification of disease-related genotypes and haplotypes, where allele frequencies in cases and controls are estimated separately and then compared [6-8]. Currently, SNPs involved in complex diseases are not well enough understood, nor are genotype data sets adequate for development of models to reliably predict disease from SNP profiles. However, the science is sufficiently advanced that studies such as presented herein can be useful to plan experiments, and to interpret data in what may well prove to be an arduous, lengthy and iterative process of identifying SNPs and SNP patterns useful as biomarkers.

Esophageal cancer, like many other cancers, has been shown to be associated with genetic as well as environmental factors that cause DNA damage. It is the sixth leading cause of cancer worldwide and seventh among American men. Among the two main types of esophageal cancer, squamous cell carcinoma and adenocarcinoma, squamous cell carcinoma accounts for about half of all esophageal cancers. Data from studies in animals suggest that oxidative damage from factors such as smoking or gastroesophageal reflux, which cause inflammation, esophagitis, and increased cell turnover, may initiate the carcinogenic process [9,10]. Studies also suggest that substantial alcohol intake, tobacco smoking and betel quid chewing increase the risk of squamous cell carcinoma [11-13].

We investigated the association of SNPs and squamous cell carcinoma of the esophagus in a case-control study of individuals from Shanxi province, a region in north-central China. Shanxi has one of the highest esophageal cancer rates in the world. The SNPs chosen for the study were a set of those associated with DNA repair, Phase I and Phase II enzymes involved in xenobiotic clearance, and with alcohol metabolism. We developed a novel adaptation of the Decision Forest pattern recognition method named Decision Forest for SNPs (DF-SNPs). The method was designated to analyze the SNPs-disease association based on the SNP data. Importantly, the DF-SNPs method utilizes the inherent differentiating ability of decision trees to separate disease and control population based on individual SNPs, the SNP types, as well as combinations of SNP types, that is, SNP patterns.

## Methods

### Study Population

Incident cases of esophageal cancer were obtained from an ongoing case-control study being conducted by the National Cancer Institute, USA and the Shanxi Cancer Institute, China. Esophageal cancer patients at the Shanxi Cancer Hospital in Taiyuan, Shanxi Province, were recruited to the case-control study. Esophageal cancer was confirmed by the pathologists at the Shanxi Cancer Hospital and National Cancer Institute and all cases were esophageal squamous cell carcinoma (ESCC). Controls matched on age, gender and neighbourhood were selected for each case. The study was approved by the Institutional Review Boards of the Shanxi Cancer Hospital and the US National Cancer Institute. After signed consent was obtained, a blood sample was collected from the participants and information on demographics and cancer risk factors including smoking, alcohol, diet, and family history were collected in an interview.

### SNP data

DNA samples were genotyped at a commercial laboratory (BioServe Biotechnologies, Ltd., Laurel, MD) by Masscode mass spectrometry genotyping. PCR primers used for genotyping were synthesized at BioServe. Oligonucleotide sequences of the primers probe can be found at . All laboratory personnel were blinded to case-control status. A total of 63 SNPs were chosen from DNA repair, Phase I, Phase II, and Alcohol metabolism related genes. The average call rate for the study was 98.5%, and duplicate analysis of three of the SNPs generated 99.7%, 99.8%, and 100% concordance. The prevalence of the variant allele ranged from 5.2% to 49.4%.

### Decision Forest for SNPs (DF-SNPs)

**Figure 1:**
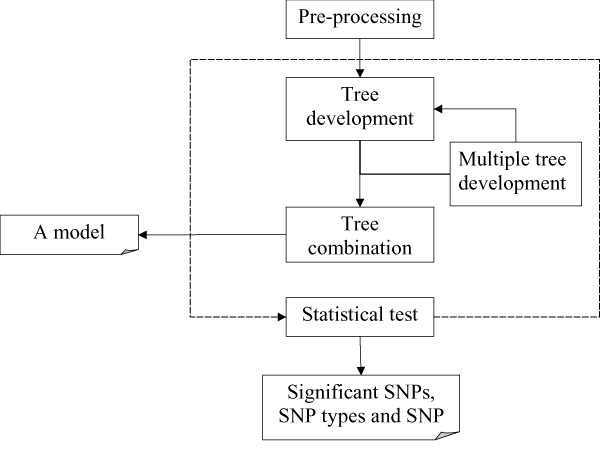
**Overview of the method of Decision Forest for SNPs (DF-SNPs). **There are several components in DF-SNPs: (1) Data pre-processing; (2) Tree development; (3) Multiple tree development; (4) Tree combination; and (5) statistical test. DF-SNPs not only produces a classifier but also identifies the significant SNPs, SNP types and SNP patterns.

DF-SNPs is an ensemble classification method that combines multiple decision trees to derive a classifier based on a fitted set of if-then rules. By combining multiple heterogeneous decision trees, DF-SNPs is effective in mitigating noise that is often prevalent in biological data, especially data from high throughput technologies, compared to single decision trees. Moreover, the algorithm is computationally inexpensive, enabling cross validation and randomization tests to readily be included. Figure 1 depicts a general flowchart of DF-SNPs. The detail information of each step in Figure 1 is described in the following sections. The DF-SNPs was developed using C.

#### Data pre-processing

It is common that the SNP types for some individuals are inconclusive using most high-throughput genotyping methods, including the Masscode mass spectrometry genotyping method. This results a data set (a spreadsheet with samples in row and SNP variables in column) that usually contains many missing values (i.e. many empty cells in the spreadsheet). Accordingly, a data pre-processing procedure is required to impute the missing data for subsequent analysis. A two-steps data pre-processing procedure was implemented in DF-SNPs. First, the samples (rows) with > 15% missing genotypes (empty cells) across all SNP variables (columns) and the SNP variables (columns) with > 15% missing genotypes (empty cells) across all the samples (rows) were removed. Next, a 10-nearest neighbor method was employed to impute the missing data. In this method, if a sample has a missing value (an empty cell) that is corresponding to a SNP variable, we identified 10 samples that not only had a measure for this SNP but also had the closest SNP profiles calculated based on the rest of the SNP variables. The genotypes of these 10 samples were used to impute the missing value by voting.

There were 1042 missing genotypes in the data set (2.9% missing values). Two SNP variables (two columns with >19% empty cells or missing genotypes) and eight samples (eight rows with >28% empty cells or missing genotypes) were removed, which resulting 1.3% missing value in the data set for imputing. The final data set for analysis after pre-processing contained 566 individuals and 61 SNPs, 391 cancer cases and 175 controls.

#### Decision Tree development

**Figure 2:**
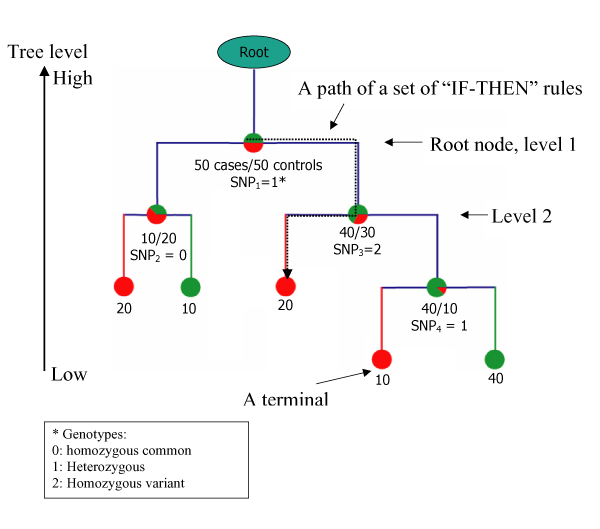
**Overview of a decision tree. **A binary tree starts from the root node. The best SNP genotype is selected at each node to separate the cases and controls using an "IF-THEN" rule. SNP types used in a path of an "IF-THEN" rule forms a SNP pattern.

A tree was developed using the S-Plus binary split method[14], where the SNPs are the independent variables grouped into three genotype categories: homozygous common, heterozygous and homozygous variant. The method started from the root node that contained all samples and identified a SNP type of all possible SNPs that divided the samples into two child nodes. Since this is a binary splitting of each tree node, the single SNP type that best divides the node populating one branch and the other two genotypes populating the other branch. The impurity (the percentage of one type of samples) of a node was measured with deviance. The process was then recursively repeated on the child nodes and the process was stopped if further splitting did not improve the purity of the child node or the node can not be further split. Figure 2 provides a hypothetical example to illustrate the tree development process. If there is a SNP data set with 50 cases and 50 controls, the process first identifies the best splitter, SNP_1 _= heterozygous, to put 70 samples in the right node (the samples with the heterozygous genotype) and 30 samples in the left node (the samples with the non-heterozygous genotype). The cases are enriched in the right node (40 to 30) compared to its parent node while the controls are enriched in the left node. In the end of tree development, the cases and controls are enriched in the different terminal nodes.

#### Multiple tree development and combination

In our previous application, multiple trees in DF used distinct independent variables to maximize their difference[15]; the variables used in one tree were not used in any other tree. Since only a limited number of SNP variables were in this application, we forced trees that selected different SNP types in splitting the root node to assure heterogeneity. That is, a SNP type selected for the root splitting in one tree was not allowed for use in splitting root nodes in other trees. However, this rule was not enforced in the subsequent splitting of the lower nodes. To ensure the comparability of trees, their qualities were maintained to the same (or close to the same) by adjusting the misclassification rate in the tree development. Finally, the classification of each sample was determined by averaging the outcomes from all trees.

#### Statistical testing

If we generate many classification models using a bootstrapping method, the relevance (or importance) of a SNP, SNP type or SNP pattern should be directly correlate to the number of times they are used by the models. Based on this assumption, we performed 2000 runs of 10-fold cross-validation and calculated the frequencies of each SNP, SNP type and SNP pattern appeared in this process. To determine the statistical significance of the findings, we also generated a null hypothesis using the same statistical procedure. This was a randomization test, where the sample classification (case or control) was randomly scrambled to generate 2000 pseudo data sets and the 10-fold cross-validation was then applied to each pseudo data set. Thus, null distributions were generated for each SNP, SNP type and SNP pattern. We then determined the critical values that corresponded to the 5% level of significance for SNPs, SNP types and SNP patterns, respectively. The statistically significant SNPs, SNP types and SNP patterns were these whose frequencies were greater than their corresponding critical values.

## Results

The study population comprised 574 individuals genotyped for 63 SNPs, of which 394 were esophageal cancer patients and 180 were age-frequencies matched controls. After removing individuals for which data was missing for more than 15% of genotypes, and removing SNP variables that were missing data for more than 15% of the individuals, the data was reduced to 566 individuals (391 cases and 175 controls) and 61 SNP variables.

**Figure 3:**
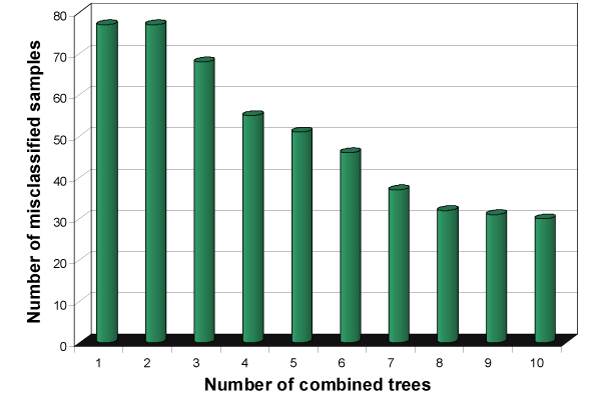
**Misclassifications versus the number of combined trees in a DF-SNPs model. **The number of combined trees correlates negatively with the misclassifications in DF-SNPs. The 10-tree model reduces the misclassifications significantly compared to the first tree.

This is a binary classification problem. The SNPs are the independent variables grouped into three genotype categories: homozygous common, heterozygous and homozygous variant. Using the DF-SNPs method, we developed a classification model to differentiate the cases from the controls. The fitted binary decision forest model thus derived contained 10 decision trees and exhibited high concordance (94.7%), sensitivity (99.0%) and specificity (85.1%), indicating that the model well differentiates disease from control samples. Figure 3 shows that the misclassifications of the model significantly diminish as trees are added to the forest, indicating that the DF method may be canceling some random variation or amplifying the nonrandom signal as trees are added. Ultimately, the 10-tree model has misclassifications of 30, much less than the 77 misclassification of the single decision tree.

The DF-SNPs algorithm includes functions that estimate on a relative basis the statistical significance of individual SNPs, SNP types and SNP patterns in differentiating SNP case-control study data. The rational behind this approach is that the frequency of a particular SNP, SNP type or SNP pattern selected by the model correlates positively with the relevance (or importance) to discriminate the cases from controls. The frequency was obtained from a multiple runs of 10-fold cross-validation procedure. To determine the statistical significance of the disease-related SNPs, SNP types and SNP patterns, a null hypothesis was first generated where the scrambled SNP data set was also subjected to the same multiple runs of 10-fold cross-validation (randomization test). Then, the frequency based on the real data set was compared with the null hypothesis. The purpose of this statistical test is to determine whether the obtained results (frequencies for a SNP, SNP type and SNP pattern) reject the hypothesis that they are merely a product of chance factors. The results of this investigation are described in the following sections.

### Identification of individual SNPs relevant to the esophageal cancer

In this section, we investigated the association of individual SNPs with the cancer. The SNPs were ranked according to frequency of use in tree node splitting in 2000 runs of 10-fold cross-validation. The ranking frequency for SNP *i *(*Freq(SNP*_*i*_*)*) is computed as:



where *SNP*_*i*_* = 1 *means that SNP *i *was used as a split rule in tree *k *at level *l *(Figure 2). *Freq(SNP*_*i*_*) *is calculated over all trees in the forest models in 2000 runs of 10-fold cross-validation. In this equation, SNPs at each lower node level are given half the frequency weight as SNPs at the preceding higher level.

An estimate of the frequency of occurrence required for a SNP to be statistically significant at 95% confidence is obtained from the random case. Specifically, for the random case, the cumulative 0.05 frequency of occurrence for SNPs rank ordered by frequency occurs for a SNP with frequency of 0.028; SNPs having frequency greater than the critical value of 0.028 are designated as significant to a 95% level of confidence. In the case-control study, nine out of total of 61 SNPs had *Freq(SNP*_*i*_*) *larger than 0.028, indicating their relevance to the esophageal cancer with P < 0.05 (Table 1).

### Identification of SNP types relevant to the esophageal cancer

In this section, we investigated the association of SNP types with the cancer. In DF-SNPs, SNP types were used to split each node in a tree, where the first split of the root node (level 1, Figure 2) selects the SNP type best separating cases from the controls. SNP types were ranked in accordance to frequency of occurrence in the first split of a tree in 2000 runs of 10-fold cross validation. In the same manner as in the preceding section for SNPs, a critical value of 0.0095 was obtained from the randomization test to correspond to 95% confidence of significance. Of 180 possible SNP types, 14 SNP types were found to have frequency of occurrence greater than the critical value (P < 0.05, Table 2). We also evaluated the odds ratio and its 95% confidence interval for SNP type, which gives an estimate of how much more likely it is that an individual with the SNP type is to be a cancer case than a control case. Of the 14, five SNP types had an odds ratio within the 95% confidence interval, indicating their significance to the esophageal cancer.

### Identification of SNP patterns relevant to the esophageal cancer

Here, we investigated the association of SNP patterns (the combination of SNP types) with the cancer. In a tree, the classification of a sample is determined by only one terminal node that is descendent from the root node through a set of "IF-THEN" rules based on *k *SNP types (Figure 2). A SNP pattern was defined as a set of SNP types used in a path of a set of the "IF-THEN" rules. We denote n-SNP pattern as a SNP pattern that contains n SNP types (n = 2, 3, 4, ...). Since the SNP types selected earlier for splitting nodes are more important than those selected later, a SNP pattern begins at the root node and the pattern is incremented as each successive node of the tree. Thus, an n-SNP pattern contains the following characteristics: (1) it always contains the SNP pattern that splits the root node; (2) the SNP types in a SNP pattern are sequentially ordered in the path of the "IF-THEN" rules; and (3) n-SNP patterns are considered to be the same if they contain the same SNP types, regardless of order.

Table 3 summarizes the analysis results of SNP patterns for up to *n *= 20. In general, the number of SNP patterns versus *n *is a distribution with fewer SNPs when *n *is either smaller or larger. When *n *is small, the number of possible SNP combinations is small. When *n *is large, it is likely that patterns contain the same SNPs, since the pattern does not depend on the order of SNP selection. The second column lists all possible SNP patterns for each n-SNP pattern. The last column gives the critical value, which corresponds to the 5% probability that a pattern's frequency could occur by chance. The third column gives the number of SNP patterns that occur with frequency above the critical value, that is, the number of SNP patterns is above the critical value with 95% confidence of statistical significance. The number of significant SNP patterns is markedly reduced compared with the number of possible SNP patterns.

We also evaluated the odds ratio and its 95% confidence interval for each n-SNP pattern, which gives an estimate of how much more likely it is that an individual with a particular SNP pattern is to be a cancer case than a control case. Results are given in Table 4 for 15 SNP patterns consisting of two SNPs (results are not reported for patterns of more than two SNPs) for which the odds ratio is within the confidence interval for distinguishing cancer cases from controls.

## Discussion

We developed a novel statistical approach, DF-SNPs, that was used for an association study between SNP type data and a case/control study of esophageal cancer. Using DF-SNPs, we identified a list of SNPs, SNP types and SNP patterns that might be associated with esophageal squamous cell carcinoma. This approach could be useful for identification of potential biomarkers based on SNP data.

We have successfully developed and used the DF method for various applications, including structure-activity relationship studies, microarray data analyses and proteomics data analyses [15-19]. Unlike previous applications of DF, the independent variables in the SNPs data set are categorical rather than continuous. Moreover, each categorical variable (SNP variable) has only three categories (three genotypes), which is a difficult problem for most classification methods. The DF-SNPs is a variant of the DF method that is specifically designed to analyze the SNP-disease association. In DF-SNPs, as in previous DF applications, multiple individual trees are combined to produce a better model. As shown in Figure 3, the DF model accuracy varies directly with the number of independent trees within the forest. The 10-tree forest that was developed has high concordance, specificity and sensitivity for the fitted data. Such a model could be used to assess the cancer potential for unknown samples solely based on the SNP profiles.

There are two important considerations for the use of DF-SNPs when compared with alternative classifier methods. First, combining identical or similar trees will not improve the quality of the forest derived from these trees and the benefit in combination can only be realized when individual trees are different or heterogeneous. Thus, each tree in DF-SNPs uses a distinct SNP type for splitting the root node, ensuring that each tree is different and encodes a different aspect of the disease-SNP association. Secondly, the individual trees of similar quality (i.e., having similar misclassification rate) when combined may cancel some of the random noise inherent in SNP type and case-control data.

The Masscode mass spectrometry-based genotyping method resulted in 3–5% missing genotypes. How to appropriately impute the missing value is important for subsequent analysis of the data generated from this technology. Accordingly, a two-step imputing method was embedded in DF-SNPs. First, we removed the individuals for whom most genotype data were missing, as well as removed SNP variables that were not detected in many individuals. Then we imputed the missing SNP genotypes for each remaining individual using a 10 nearest neighbor method. This approach proved to be efficient for preprocessing the SNPs data set.

In DF-SNPs, the potential cancer-related SNPs, SNP types and SNP patterns were identified on the basis of frequencies of occurrence in decision tree splitting for all trees during 10-fold cross-validation. A randomization test was also done with cross-validation to provide a random distribution of frequencies for comparison with the fitted model. Comparison of the fitted and random frequencies provided the estimates of the statistical significance of SNPs, SNP types and SNP patterns in distinguishing cases versus controls

To investigate the relevant SNPs to the esophageal squamous cell carcinoma, we employed a weighted approach to calculate the frequency of each SNP. Given the fact that the SNPs used for splitting the root node are applied to the entire data set while those used in the next split at the second level are applied to a much smaller portion of the data set (normally around the half of the data set), and that subsequent splits are applied to even smaller numbers, the relevance of the SNPs to cancer should decrease proportionally to the height of the tree level where they were selected. We compared several weighted factors by taking into account of the tree level to calculate the frequency of SNPs, including 1, 1.25, 1.5 and 2. Since other weighted factors potentially eliminated the SNPs used in the root node (results not shown), the weighted factor of 2 was selected, indicating that the relevance (or importance) of a SNP is reduced by half as moves to each subsequent lower level.

The odds ratios and corresponding confidence intervals were used to identify 14 SNP types that distinguish cases from controls at 95% confidence (Table 2). Of these, five had confidence intervals that were either >1 or <1 and thus are likely to be more significant. Of the five, two had confidence intervals <1, indicating their possible association with reduced cancer potential. Three with confidence interval >1 are indicated to be associated with increased cancer risk. We further found that two GADD45B E1122 genotypes (numbers 1 and 4 in Table 2) are suggested to modify cancer risk differently, with the homozygous common genotype possibly increasing esophageal cancer risk and the heterozygous genotype possibly decreasing cancer risk. These data suggest a potentially important role for polymorphisms of GADD45B E1122 as a biomarker of esophageal cancer risk.

Prospectively, given appropriate and sufficient data, DF-SNPs provides a methodology that could identify the possible SNP-SNP associations, that is, SNP patterns involved in genetic-based variation in cancer risk. Table 4 illustrates how such predictions would appear for the case of patterns of two SNPs. Of the 15 2-SNP patterns in Table 4, it is interested that the data suggests that 12 are associated with decreased risk and two are associated with increased risk. Also notable is that odds ratios are substantially larger for the patterns of two SNPs than for individual SNPs (compare Table 2 with Table 4), possibly indicating that patterns of SNPs are more predictive of cancer risk than individual SNPs. Not surprisingly, analysis showed that odds rations vary in direct proportion to the length of SNP patterns (results not shown).

## Conclusion

Several statistical approaches including logistic regression methods have been used to analyze case/control SNP data. In this article, we propose DF-SNPs as method to analyze SNPs data for purposes of biomarker identification. DF-SNPs is a novel variant of DF that was previously developed in our lab. DF-SNPs was specifically structured to deal with the three genotype categories of SNP data. The DF-SNPs algorithm incorporates the following processes: (1) a two-step approach to impute missing SNPs; (2) use of 10-fold cross-validation when calculating frequency of SNPs, SNP types and SNP patterns in discriminating cases from controls; and (3) estimating statistical significance by comparing frequencies from (2) with those from a random test also using 10-fold cross validation. Using DF-SNPs, potential biomarkers could be quickly identified based on SNPs, SNP types or SNP patterns. This method complements other methods currently in use.

## Authors' contributions

QX developed the method presented in the paper and wrote the first draft manuscript. WT guided the method development and helped writing the manuscript. HH developed the missing data imputing method and also involved the DF-SNPs development. LR generated the SNP data set used in this study. RP helped writing the manuscript. LR generated the SNP data set used in this study. ZZT, NH and PRT conducted the case-control study. All authors read and approved the final manuscript.

## Figures and Tables

**Table 1:** Statistically significant SNPs (P < 0.05) relevant to esophageal squamous cell carcinoma. In 2000 runs of 10-fold cross-validation, nine SNPs were found to be the esophageal squamous cell carcinoma relevant. They had frequencies greater than the critical value of 0.028 at the 5% level of significance (P < 0.05).

No	SNPs		Frequency	Gene function
			
	Gene	RS number		
1	GADD45B	E1122	0.186	DNA repair
2	NQO1	rs1800566	0.069	Phase II
3	adh1b_55		0.064	Alcohol-related
4	ERCC5	rs17655	0.064	DNA repair
5	COMT	rs4818	0.052	Phase II
6	GADD45B	rs14384	0.049	DNA repair
7	ercc5_55		0.048	DNA repair
8	CYP1A1	rs1048943	0.035	Phase I
9	GPX1_	rs1800668	0.030	Phase II

RS – Reference SNP

**Table 2:** Statistically significant SNP types relevant to esophageal squamous cell carcinoma. In 2000 runs of 10-fold cross-validation, 14 SNP types are found to be the esophageal squamous cell carcinoma relevant. They had frequencies greater than the critical value of 0.0095 at the 5% level of significance (P < 0.05). By applying these SNP types to the SNP data set, the first five SNP types have 95% confidence intervals (CIs) whose upper and lower limits not cross with OR = 1.

No	OR	95% CI	SNP site		
			
			Gene	RS number	Genotype
1	1.66	1.16 – 2.37	GADD45B	E1122	Homozygous common
2	1.50	1.05 – 2.15	ercc5_55		Homozygous common
3	1.44	1.00 – 2.06	COMT	rs4818	Homozygous common
4	0.55	0.38 – 0.79	GADD45B	E1122	Heterozygous
5	0.45	0.23 – 0.87	NQO1	rs1800566	Homozygous variant

6	1.64	0.70 – 3.87	ERCC5	rs17655	Homozygous variant
7	1.57	0.76 – 3.26	GPX1	rs1800668	Homozygous variant
8	1.43	0.98 – 2.09	NQO1	rs1800566	Heterozygous
9	1.05	0.73 – 1.52	ERCC2	rs1052559	Heterozygous
10	1.00	0.51 – 1.97	GADD45B	rs14384	Homozygous variant
11	0.99	0.51 – 1.91	CYP1A1	rs1048943	Homozygous variant
12	0.87	0.60 – 1.26	cyp1a2_5		Heterozygous
13	0.83	0.57 – 1.20	CYP1A2	rs2472304	Heterozygous
14	0.59	0.27 – 1.32	adh1b_55		Heterozygous

**Table 3:** n-SNP patterns identified by DF-SNPs (n = 1, 2, ...,20).

Pattern Length	Total number of patterns	Number of patterns above the critical value	Number of patterns with differentiating ability	Critical Value
1	72	14	5	0.0095223
2	578	52	15	0.0002364
3	5558	3560	379	0.0000375
4	28562	5015	204	0.0000180
5	88124	10898	169	0.0000119
6	175401	15529	59	0.0000095
7	255460	16639	8	0.0000088
8	291469	14217	0	0.0000094
9	265793	9738	0	0.0000117
10	205505	4512	0	0.0000173
11	138306	1853	0	0.0000291
12	81279	560	0	0.0000536
13	41785	241	0	0.0001036
14	18557	70	0	0.0002274
15	7047	40	0	0.0005389
16	2436	16	0	0.0013277
17	735	15	0	0.0031564
18	180	17	0	0.0092945
19	38	11	0	0.0300700
20	9	3	0	0.0997076

**Table 4:** Statistically significant 2-SNP patterns relevant to esophageal squamous cell carcinoma. In 2000 runs of 10-fold cross-validation, 52 2-SNP patterns were found to be esophageal cancer relevant. They had frequencies greater than the critical value of 0.00024 at the 5% level of significance (P < 0.05). By applying these 2-SNP patterns to the SNP data set, 15 2-SNP patterns have 95% confidence intervals (CIs) whose upper and lower limits not cross with OR = 1.

No	OR	95% CI	SNP site 1			SNP site 2		
			
			Gene	RS number	Genotype	Gene	RS number	Genotype
1	2.33	1.29 – 4.19	NQO1	rs1800566	Heterozygous	GADD45B	E1122	Homozygous common
2	2.09	1.41 – 3.09	GADD45B	E1122	Homozygous common	COMT	rs4818	Homozygous common
3	2.03	1.37 – 3.00	ercc5_55		Homozygous common	GADD45B	E1122	Homozygous common
4	0.61	0.38 – 0.98	cyp1a2_5		Heterozygous	GADD45B	E1122	Heterozygous
5	0.60	0.39 – 0.94	GADD45B	E1122	Heterozygous	ERCC2	rs1799787	Heterozygous
6	0.60	0.38 – 0.94	GADD45B	E1122	Heterozygous	COMT	rs4818	Heterozygous
7	0.58	0.37 – 0.92	GADD45B	E1122	Heterozygous	CYP1A2	rs2472304	Heterozygous
8	0.58	0.37 – 0.89	GSTM3	rs1537234	Heterozygous	GADD45B	E1122	Heterozygous
9	0.57	0.36 – 0.90	GADD45B	E1122	Heterozygous	COMT	rs4818	Homozygous common
10	0.57	0.37 – 0.87	LIG1	rs20579	Heterozygous	GADD45B	E1122	Heterozygous
11	0.54	0.35 – 0.84	GADD45B	E1122	Heterozygous	ERCC2	rs1052559	Homozygous common
12	0.45	0.28 – 0.72	GADD45B	rs14384	Homozygous common	GADD45B	E1122	Heterozygous
13	0.40	0.26 – 0.63	GADD45B	E1122	Heterozygous	ERCC5	rs17655	Homozygous common
14	0.22	0.06 – 0.73	adh1b_55		Homozygous variant	GADD45B	E1122	Heterozygous
15	0.16	0.04 – 0.62	NQO1	rs1800566	Homozygous variant	GADD45B	E1122	Homozygous common
